# Association of Surgical Experience With Risk of Complication in Total Hip Arthroplasty Among Patients With Severe Obesity

**DOI:** 10.1001/jamanetworkopen.2021.23478

**Published:** 2021-09-01

**Authors:** Alexander Charalambous, Daniel Pincus, Sasha High, Fok-Han Leung, Suriya Aktar, J. Michael Paterson, Donald A. Redelmeier, Bheeshma Ravi

**Affiliations:** 1 Division of Orthopaedic Surgery, Sunnybrook Health Sciences Centre, Toronto, Ontario, Canada; 2 Division of Orthopaedic Surgery, Department of Surgery, University of Toronto, Toronto, Ontario, Canada; 3 Faculty of Medicine, University of Toronto, Toronto, Ontario, Canada; 4 ICES (formerly Institute for Clinical Evaluative Sciences), Toronto, Ontario, Canada; 5 Institute of Health Policy, Management, and Evaluation, University of Toronto, Toronto, Ontario, Canada

## Abstract

**Importance:**

Severe obesity is a risk factor for major early complications after total hip arthroplasty (THA).

**Objective:**

To determine the association between surgeon experience with THA in patients with severe obesity and risk of complications.

**Design, Setting, and Participants:**

This retrospective population-based cohort study was performed in Ontario, Canada, from April 1, 2007, to March 31, 2017, with data analysis performed from March 2020 to January 2021. A cohort of patients who received a primary THA for osteoarthritis and who also had severe obesity (body mass index [calculated as weight in kilograms divided by height in meters squared] ≥40) at the time of surgery was defined. These patients were identified using the Canadian Institute for Health Information Discharge Abstract Database and physician claims from the Ontario Health Insurance Plan. Generalized estimating equations were used to determine the association between overall THA and severe obesity–specific THA surgeon volume and the occurrence of complications after controlling for potential confounders. The study hypothesized that surgeon experience specific to patients with severe obesity could further reduce the risk of complications.

**Exposures:**

Primary THA.

**Main Outcomes and Measures:**

Complications were considered as a composite outcome (revision, infection requiring surgery, or dislocation requiring reduction), within 1 year of surgery. This was defined before the study, as was the study hypothesis.

**Results:**

A total of 4781 eligible patients was identified. The median age was 63 (interquartile range [IQR], 56-69) years, and 3050 patients (63.8%) were women. Overall, 186 patients (3.9%) experienced a surgical complication within 1 year of surgery. The median overall THA surgeon volume was 70 (IQR, 46-106) cases/y, whereas the median obesity-specific surgeon volume was 5 (IQR, 2-9) cases/y. After controlling for patient and hospital factors, greater obesity-specific THA surgeon volume (adjusted odds ratio per additional 10 cases, 0.65 [95% CI, 0.47-0.89]; *P* = .007), but not greater overall THA surgeon volume (adjusted odds ratio per 10 additional cases, 0.97 [95% CI, 0.93-1.02]; *P* = .24), was associated with a reduced risk of complication.

**Conclusions and Relevance:**

Increased surgeon experience performing THA in patients with severe obesity was associated with fewer major surgical complications. These findings suggest that surgeon experience is required to mitigate the unique anatomical challenges posed by surgery in patients with severe obesity. Referral pathways for patients with severe obesity to surgeons with high obesity-specific THA volume should be considered.

## Key Points

**Question:**

Is there an association between surgeon experience with total hip arthroplasty (THA) in patients with severe obesity and the risk of complications?

**Findings:**

In this population-based cohort study of 4781 patients, volume of obesity-specific procedures was found to be associated with fewer major surgical complications after THA performed in patients with severe obesity (reduction in risk by 35% for every 10 additional patients).

**Meaning:**

These findings suggest that referral pathways to surgeons with high obesity-specific THA volume should be considered for this group of patients.

## Teaser

This cohort study assesses whether surgeon experience with total hip arthroplasty (THA) in patients with severe obesity is associated with risk of complications.

## Introduction

Obesity is a risk factor for major complications after numerous surgical procedures, including total joint arthroplasty (TJA).^1,2,3,4,5^ Class 3 or severe obesity (defined as a body mass index [BMI; calculated as weight in kilograms divided by height in meters squared] of ≥40) in particular is associated with increased risk for surgical site infection, dislocation, early loosening of prostheses, revision surgery, periprosthetic fracture, and thromboembolism after TJA.^6,7,8,9^ At the same time, the demand for TJA among patients with obesity continues to increase.^10^

Despite the higher risk of complications, patients with obesity also experience similar improvements in function and quality of life after TJA.^6,7,11^ To mitigate the risk of complications, many physicians and surgeons encourage weight loss in patients with severe obesity before recommending TJA. In fact, several hospitals in the US have set a cutoff BMI of 40, above which they will refuse to offer arthroplasty.^12^ However, sufficient weight loss before surgery is not always possible, particularly when patients with obesity also have end-stage arthritis that makes regular exercise impractical. Unfortunately, refusal to perform semielective procedures (such as joint replacement) in patients with severe obesity may perpetuate the cycle of weight bias and discrimination these patients experience within health care systems.^13,14^

The higher risk for postoperative complications in patients with severe obesity can be associated with comorbidities, such as type 2 diabetes, that are potentially modifiable and may be optimized before elective surgery.^15^ However, severe obesity also leads to nonmodifiable anatomical challenges that make procedures more complicated (eg, adequate exposure, retractor placement, component positioning) that may contribute to increased complication risk.^15^ These technical surgical challenges may in turn be mitigated by increased surgeon experience in dealing specifically with such challenges in this patient population. Although the association between surgeon volume and patient outcomes is well-established for TJA,^16,17,18,19^ we hypothesized that surgeon experience specifically with patients with severe obesity could further reduce the risk of complications.

## Methods

### Study Sample

We defined a cohort of consecutive adults (>18 years of age) with severe obesity in Ontario, Canada, who received their first primary elective total hip arthoplasty (THA) for osteoarthritis from April 1, 2007, to March 31, 2017. The data were obtained retrospectively and analyzed from March 2020 to January 2021. Ontario has a solely public health care system. There is no private coverage for THA, making it feasible for us to identify all hip replacements and subsequent complications treated throughout the province. Use of the data in this study was authorized under section 45 of Ontario’s Personal Health Information Protection Act, which does not require review by a research ethics board or informed consent. This study followed the Strengthening the Reporting of Observational Studies in Epidemiology (STROBE) reporting guideline.

### Data Sources

We used hospital discharge abstracts from the Canadian Institute for Health Information Discharge Abstract Database and physician claims from the Ontario Health Insurance Plan (OHIP). We identified patients using specific procedure codes recorded in hospital discharge abstracts (Canadian Classification of Health Interventions code 1VA53LA) and OHIP claims (fee code R440). Patient demographics, including age and sex, were obtained from the OHIP Registered Persons Database^20,21,22^ using the same validated definitions as the Ontario Ministry of Health and Long-Term Care.^23^ Patients with severe obesity were defined as those with a BMI of 40 or greater at the time of surgery as identified by a supplementary OHIP billing code added by the operating surgeon (E676, which denotes BMI ≥40). The ICES (formerly Institute for Clinical Evaluative Sciences) is governed by the Personal Health Information Protection Act and is responsible for storing and managing this data. Previous validation studies have identified a high positive predictive value and sensitivity of Ontario administrative codes in other surgical specialties.^24,25,26^

### Surgeon Overall and Obesity-Specific Volumes

For each THA case, annual surgeon overall volume was defined as the number of primary or revision THA procedures performed by the surgeon in the 365 days before the procedure (regardless of whether the patient had severe obesity). The annual surgeon obesity-specific volume was defined as the number of primary or revision THA procedures the surgeon performed for patients with a BMI of 40 or greater during the same period. The intent was to distinguish general experience from obesity-specific experience.

### Primary Outcome: Major Surgical Complication

The primary study outcome was the occurrence of a major surgical complication (revision arthroplasty, infection requiring additional surgery, or dislocation) within 1 year of surgery. Revision procedures were identified using *International Statistical Classification of Diseases and Related Health Problems, Tenth Revision, Canada* (*ICD-10-CA*)/Canadian Classification of Health Interventions procedure codes accompanied by the supplementary status attribute R with an appropriate surgeon billing code. Infections requiring surgery were identified by the first occurrence of an *ICD-10-CA* diagnostic code for intra-articular infection, with a confirmatory code for an irrigation and debridement, or an OHIP fee code for a spacer insertion. Dislocations were identified by the presence of a procedure code for closed/open hip reduction.

### Covariates of Interest

We measured and controlled for several factors associated with the occurrence of complications after hip replacement. These included patient age, sex, socioeconomic status, and comorbidities. Comorbidities listed on hospital discharge abstracts in the 3 years preceding the index THA admission were categorized according to an adaptation of the Deyo-Charlson Comorbidity Index.^27^ The Johns Hopkins ACG System (version 10) was used to identify patients as frail based on diagnosis codes on hospital discharge abstracts and physician service claims in the 2 years preceding the index THA admission.^28^ The presence of diabetes was identified using a validated algorithm.^29^ Neighborhood household income quintile was used as a surrogate measure for socioeconomic status and living conditions.^30,31^

### Statistical Analyses

Spearman rank correlation coefficients were used to quantify the association between surgeon obesity-specific THA volume and surgeon overall THA volume. Restricted cubic splines with 3 knots were used to visualize the association between surgeon obesity-specific THA volume and the risk for complications. Generalized estimating equations were used to test the association of surgeon obesity-specific THA volume with the risk for complications after controlling for patient age, sex, comorbidities, income quintile, hospital volume, and the year the procedure was performed, in addition to controlling for clustering of patients within surgeons. This multivariate analysis was then repeated after replacing the obesity-specific THA volume with surgeon overall THA volume. All analyses were performed at ICES using SAS, version 9.3, for UNIX (SAS Institute, Inc). The type I error probability was set to 0.05 for all analyses. Two-sided *P* < .05 indicated statistical significance.

## Results

### Patient Characteristics

From April 1, 2007, to March 31, 2017, we identified 4781 eligible THA recipients with a median age of 63 (interquartile range [IQR], 56-69) years. Of these, 3050 patients (63.8%) were female and 1731 (36.2%) were male. All the included patients were classified as having severe obesity at the time of surgery, and procedures were performed by 317 surgeons across 81 hospitals. One thousand five hundred ninety-five patients in our cohort (33.4%) underwent surgery at an academic center. Whereas most patients (3215 [67.2%]) had a Deyo-Charlson Comorbidity Index of 0, 1590 (33.3%) had diabetes and 313 (6.5%) were classified as frail. A total of 186 patients (3.9%) had a major surgical complication within 1 year of surgery (Table 1).

**Table 1.** Characteristics of Eligible THA Recipients and Associated Complications

Characteristic	Patient data (n = 4781)^a^
Demographic	
Age, median (IQR), y	63 (56-69)
Female	3050 (63.8)
Male	1731 (36.2)
Income quintile	
1 (lowest)	929 (19.5)
2	1032 (21.6)
3	957 (20.1)
4	918 (19.2)
5 (highest)	937 (19.6)
Comorbidities	
Diabetes	1590 (33.3)
Frailty^b^	313 (6.5)
Deyo-Charlson Comorbidity Index	
0	3215 (67.2)
1	979 (20.5)
2	377 (7.9)
≥3	210 (4.4)
Admission characteristics	
Teaching hospital	1595 (33.4)
Hospital volume, median (IQR)	304 (193-515)
Surgeon overall THA volume, median (IQR)	70 (46-106)
Surgeon severe obesity–specific THA volume, median (IQR)	5 (2-9)
Complications	
Major surgical complication	186 (3.9)
Revision	118 (2.5)
Infection	142 (3.0)
Dislocation	29 (0.6)

Abbreviations: IQR, interquartile range; THA, total hip arthroplasty.

^a^ Unless otherwise indicated, data are expressed as number (%) of patients. Owing to missing data, numbers may not total 4781 for each characteristic.

^b^ Definition based on the Johns Hopkins ACG System (version 10).

### Surgeon Volume

**Figure 1.**
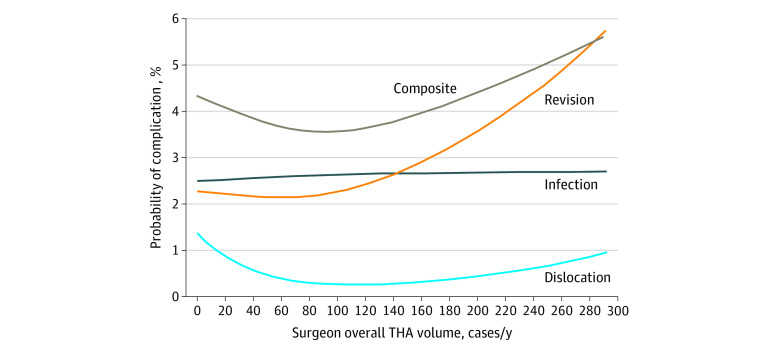
Probability of Complications After Total Hip Arthroplasty (THA) in Patients With Severe Obesity by Surgeon Overall THA Volume

**Figure 2.**
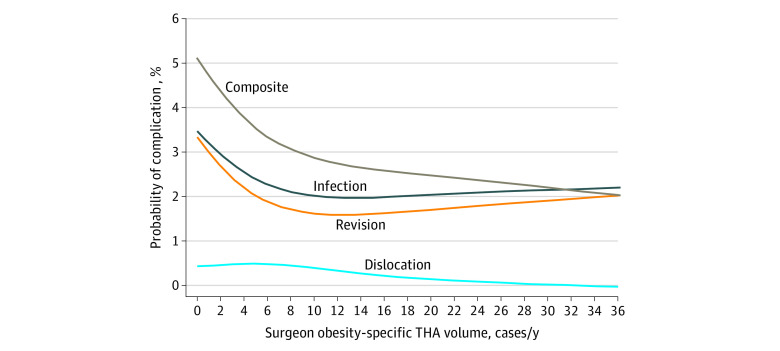
Probability of Complications After Total Hip Arthroplasty (THA) in Patients With Severe Obesity by Surgeon Obesity-Specific THA Volume

The median overall THA surgeon volume was 70 (IQR, 46-106) cases/y, whereas the median obesity-specific THA surgeon volume was 5 (IQR, 2-9) cases/y, with only a moderate correlation between the two (Spearman rank correlation coefficient, 0.54). Of the 4781 patients in the cohort, 4103 (85.8%) received their surgery from a high-volume surgeon. The splines used to correlate overall THA volume and complications within 1 year of surgery did not suggest an association between volume and outcome for patients with severe obesity (Figure 1). In contrast, the splines created to identify an association between obesity-specific THA surgeon volume and complications suggested very high complication rates at low volumes, with a steep decline until the volume neared approximately 10 cases/y, after which the apparent improvement for each additional procedure leveled off (Figure 2).

### Multivariate Analysis

After controlling for patient age, sex, comorbidities, income quintile, hospital volume, procedure year, and clustering of patients within surgeons, obesity-specific procedure volume was found to be protective for major surgical complications after THA performed in patients with obesity, with a decline in complications of approximately 35% for every 10 cases (adjusted odds ratio per additional 10 cases, 0.65 [95% CI, 0.47-0.89]; *P* = .007). In contrast, greater overall THA volume was not associated with occurrence of complications among patients with morbid obesity who were receiving THA (adjusted odds ratio per 10 additional cases, 0.97 [95% CI, 0.93-1.02]; *P* = .24) (Table 2).

**Table 2.** Multivariate Analysis of Risk for Major Surgical Complication

Characteristic	Adjusted OR (95% CI)	*P* value
Age	1.01 (0.99-1.03)	.27
Male sex	0.74 (0.54-1.03)	.08
Income quintile		
1	1.00 (0.62-1.62)	>.99
2	1.06 (0.67-1.66)	.81
3	0.83 (0.49-1.40)	.48
4	0.77 (0.48-1.23)	.27
5	1 [Reference]	NA
Deyo-Charlson Comorbidity Index		
0	1 [Reference]	NA
1	0.78 (0.52-1.18)	.24
2	1.22 (0.71-2.08)	.48
≥3	0.54 (0.22-1.31)	.17
Diabetes	1.06 (0.77-1.45)	.72
Frailty^a^	2.31 (1.46-3.67)	<.001
Hospital volume (cases/y)	1.00 (1.00-1.00)	.92
Surgeon severe obesity–specific THA volume (per additional 10 cases/y)	0.65 (0.47-0.89)	.007
Surgeon overall THA volume (per additional 10 cases/y)	0.97 (0.93-1.02)	.24

Abbreviations: NA, not applicable; OR, odds ratio; THA, total hip arthroplasty.

^a^ Definition based on the Johns Hopkins ACG System (version 10).

## Discussion

In a cohort of patients with severe obesity (BMI ≥40) undergoing elective primary THA, greater surgeon experience performing THA specifically in patients with severe obesity was associated with reduced risk of surgical complications. The association was curvilinear, with the improvement in outcomes associated with increased experience leveling off at approximately 10 cases/y. Among the 3.9% of patients who experienced a serious complication within 1 year of their THA, this risk declined by approximately 35% for every 10 additional obesity-specific THA procedures performed by the surgeon in the year before the index surgery. To our knowledge, this study is the first to demonstrate this nuance of surgeon experience specifically in patients with severe obesity.

Patients with severe obesity were treated by surgeons with higher median annual volumes relative to the general population,^16^ suggesting that the former patients are triaged to higher-volume surgeons. However, although surgeons who perform more THAs overall have been shown to have better outcomes,^16^ we did not observe a protective association of overall surgeon experience on outcomes in patients with obesity. These findings indicate that specific experience performing THA in patients with severe obesity appears to be advantageous in preparing surgeons for specific operative challenges of these cases. The same principle may generalize to other types of complex primary THAs whereby, rather than overall volume, specific experience performing THA for dysplasia, acetabular protrusio, spinopelvic fusion, femoral neck fractures, and posttraumatic arthritis leads to improved outcomes in these situations. Future work may assess this notion, and although meaningful specific experience in some of these subgroups may be difficult to achieve, the prevalence of severe obesity among recipients of THA in North America continues to increase.^10^

Weight bias and discrimination can be an ever-present part of the health care experience for patients with severe obesity, and this includes the provision of semielective procedures such as hip replacement.^32,33,34^ These patients have increased challenges being referred for surgical care, and once seen by a surgeon are more likely to have their surgery delayed pending weight loss.^34^ Although obesity has been described as a modifiable factor in patients requiring joint replacement,^35,36^ this is often not the case, particularly because severe arthritis often precludes meaningful physical activity. We should note, however, that diet and calorie control is the most important element in weight loss and should be encouraged in patients with obesity.^37^ When we consider the role of exercise, it has also been shown to play a key role, with a combination of diet and exercise being the most effective in weight loss and improvement of cardiovascular risk factors.^37^ In addition, obesity remains underdiagnosed and undertreated in many countries, with many patients finding it difficult to access care.^38^ As a result, patients may feel trapped between needing surgery to help increase their activity and not getting the support they need to lose weight to qualify. One approach to minimizing complications in patients with severe obesity might be to triage their care to surgeons with specific experience in operations for patients with severe obesity. Patients with obesity have been shown to have lower functional scores at baseline and postoperatively compared with patients without obesity.^11^ We must therefore continue to advocate weight loss where possible preoperatively.

Surgery in patients with severe obesity, including joint replacement, poses unique anatomical and technical challenges. Although we expected most patients with severe obesity would have had their surgery at high-volume teaching hospitals, only 1595 patients in our cohort (33.4%) underwent surgery at an academic center, with the remainder undergoing surgery at community hospitals. We also anticipated that most patients with severe obesity would have their THA performed by high-volume surgeons (generally defined as >35 cases/y), and indeed, 4103 patients in our cohort (85.8%) received their surgery from a high-volume surgeon. However, the benefit conferred by general experience was not observed in patients with severe obesity. One reason for this may be that the median number of THAs in patients with severe obesity performed annually was still quite rare (5 cases/y).

The high complication rate and cost to the health care system associated with performing THA in patients with severe obesity warrant initiatives to ensure consistent and appropriate care for these complex patients.^1^ Severe obesity presents surgeons with nonmodifiable anatomical challenges that make THA more complicated and contribute to increased complication risk in these cases. In this study, we identified increased surgeon experience in dealing specifically with the challenges in this patient population as a factor associated with reduced complications. Formal policy and referral networks that specify that these complex cases be completed by experienced surgeons may also be helpful. Patients with severe obesity may be better served undergoing THA by surgeons specifically experienced with performing THA in this patient population. It has been suggested previously that bariatric surgery before TJA is another potential solution to reduce postoperative complications.^39^ However, more recent literature reviews^40,41,42^ have suggested that bariatric surgery before TJA does not significantly reduce the risk of postoperative complications, including infection or revision rate. We therefore must continue to explore avenues such as referral networks and specialist hubs to help reduce complications in this group of patients.

### Strengths and Limitations

The strengths of our study include the use of population-based health administrative data to assemble a large sample of patients with severe obesity who received a hip replacement. We also considered patient, hospital, and surgeon factors associated with major complications.

Our study has some limitations. First, we did not have information on patient-reported outcomes, including post-THA pain, functioning, and improvements in quality of life. Thus, we do not know whether surgeon experience with severe obesity is also associated with these outcomes. However, because complications are associated with worse patient-reported outcomes, it is likely that greater surgeon experience with severe obesity also contributes to improved patient-reported outcomes in this population. Second, we were unable to adjust for type of implant, use of bone cement, and other technical aspects of the procedure that are associated with complication rates after THA. Surgeons with greater experience with severe obesity may systematically differ from those with less experience with respect to these techniques, and that in turn may contribute to the differential results. However, one could argue that these differences are themselves representative of the surgeons’ increased experience performing THA in patients with severe obesity. Last, our study does not account for an accumulative value of surgeon experience during the period of the study and focuses only on the preceding 365 days. Prior work has shown that increased surgeon experience over time, as represented by surgeon age, is not associated with improved patient outcomes.^16^ In the present study, however, surgeon volume was associated with improved patient outcomes. We acknowledge that previous work has not assessed outcomes in patients with obesity, but we still believe that recent surgeon volume is a valid marker of surgeon experience.

## Conclusions

Among patients with severe obesity who underwent elective primary THA, those patients with procedures performed by surgeons who had performed a greater number of THA procedures in patients with severe obesity experienced fewer complications. Our findings indicate that overall experience performing THA is not sufficient to optimize outcomes for patients with severe obesity undergoing this procedure. Instead, patients with severe obesity who undergo THA may be better served by surgeons who are specifically experienced with performing THA in this patient population. To optimize patient outcomes, our results suggest referring physicians should preferentially consider surgeons with the most experience performing this procedure in individuals with severe obesity. In larger population centers, centralized referral hubs or centers of excellence are potential solutions. In addition, mentorship networks of orthopedic surgeons could nurture and enhance capacity to provide care for patients with severe obesity who require THA.
